# 

**Published:** 1949-07

**Authors:** 


### Local Medical Notes

ON 10th April, General Sir Ronald Adam opened the new library and reading room at Southmead Hospital. The opener appeared to believe this was the first time such an amenity had ever been provided : in addition to the reading room there is a service for delivering books in the wards. Southmead is now as well off as many older hospitals in this respect.

The Bristol Medical Reunion Dinner was held at the University Union (Victoria Rooms) on 20th April. Sir Lionel Whitby, C.V.O., M.C., M.D., F.R.C.P., was the guest of honour and 160 medical men and women were present. The arrangements were in the experienced hands of Dr. R. C. Clarke : good eating, *very* good drinking and reunion of old friends produced a pleasant and memorable occasion.

### APPOINTMENTS

**Order of the Hospital of St. John of Jerusalem.**—*Officers*, A. P. Gorham, M.B.; *Serving Brothers*, J. J. Giraldi, O.B.E., M.R.C.S., Surgeon Commander J. L. S. Coulter, D.S.C., M.R.C.S.

**Medical Officer of Health for Gloucestershire.**—G. F. Bramley, M.D., D.P.H.

**United Bristol Hospitals.**—

J. H. Middlemiss, M.D., D.M.R.D., F.F.R., Director of the Radiodiagnostic Department.

Robert P. Warin, M.D., M.R.C.P., Dermatologist.

### UNIVERSITY OF BRISTOL

### MEDICAL PRIZES AND SCHOLARSHIPS, 1948–49

*Thomas Francis Edgeworth and Francis Henry Edgeworth Prize.*—N. G. Sanerkin.

*Edward Fawcett Memorial Prize.*—F. J. Applebe and N. G. Sanerkin, jointly.

*Foyle Prize.*—N. G. Sanerkin.

*Mackie Prize.*—Mr. G. Walters.

*Russell Cooper Prize.*—Mr. E. A. Houghton.

*Lady Haberfield Scholarship.*—Miss D. Ellis.

*Marshall and Clarke Prize.*—Mr. E. A. Houghton.

*Sanders Scholarship.*—Mr. J. D. Sheward.

*Henry Clarke Prize.*—Mr. P. C. Farrant.

*Medical Suple Prize.*—Mr. J. D. Sheward.

*Surgical Suple Prize.*—Mr. D. M. Gambier.

*Tibbits Memorial Prize.*—Mr. H. Schnieden.

*Barrett Roué Prize.*—Mr. H. Schnieden.

*Augustin Prichard Prize.*—Mr. B. B. Beeson.

### DEGREES AND DIPLOMAS

**University of Bristol.**—

M.B., CH.B. (*with Second Class Honours*): D. M. Gambier (with Distinction in Public Health), June M. Smith (with Distinction in Obstetrics). *Pass:* Joan H. Barnes (with Distinction in Public Health), B. B. Beeson, D. J. Brain, Joan K. Cheeseman, Jennifer M. Comely, P. C. Farrant, Elizabeth M. Fraser, Patricia M. Gilbert, Pamela M. Hellings, Sybil E. Jacob, C. D. Linton, J. P. Martin, Elizabeth M. Morgan, June Morgan, P. A. Normandale, J. S. R. R. Old, H. Schneiden, J. D. Sheward (with Distinction in Medicine), Medora Storkey, M. P. Walker, G. Walters, Olwen J. West, Catherine C. White.

M.B., CH.B.—*Second Examination:* N. G. Blackwell, R. W. Digby, S. T. Guest, M. E. Jackman, Marguerite A. R. King, R. D. Lalloo, Sheila A. Martyn, S. E. Mbanefo, J. H. Perry, W. E. Smith, D. W. G. Stone, J. T. Wheeler.

D.P.H.—K. G. Bergin, Peggy Beynon, D. W. Browne, J. E. Krzywiec, J. D. Medhurst.

D.P.M.—I. M. Davies, I. A. Macdonald.
D.M.R.D.—J. H. E. Bergin, W. R. Cole.
D.M.R.T.—K. Lal, H. H. Robinson.
B.D.S.—L. M. Lloyd.
L.D.S.—J. T. Sanders, H. Sheffield.

**University of Cambridge.**—M.B., B.CH. : E. J. Watson-Williams.

**Royal College of Surgeons.**—F.R.C.S.—H. K. Bournes, T. O. Candler, J. H. Peacock, H. Wood.

**Conjoint Board.**—M.R.C.S., L.R.C.P.—E. G. Clarkson, Patricia M. Gilbert, K. N. J. Pocock, J. M. Smith, Medora Storkey.

